# Gaps in Indigenous disadvantage not closing: a census cohort study of social determinants of health in Australia, Canada, and New Zealand from 1981–2006

**DOI:** 10.1186/1471-2458-14-201

**Published:** 2014-02-25

**Authors:** Francis Mitrou, Martin Cooke, David Lawrence, David Povah, Elena Mobilia, Eric Guimond, Stephen R Zubrick

**Affiliations:** 1 Telethon Institute for Child Health Research, The University of Western Australia, PO Box 855 West, Perth, WA 6872, Australia; 2 School of Public Health and Health Systems & Department of Sociology and Legal Studies, University of Waterloo, Waterloo, ON N2L 3G1, Canada; 3 Strategic Research Directorate, Aboriginal Affairs and Northern Development Canada, Gatineau, Québec, Canada; 4 Department of Sociology, University of Western Ontario, London, ON, Canada

**Keywords:** Indigenous, Inequality, Social determinants of health, Education, Unemployment, Income

## Abstract

**Background:**

Australia, Canada, and New Zealand are all developed nations that are home to Indigenous populations which have historically faced poorer outcomes than their non-Indigenous counterparts on a range of health, social, and economic measures. The past several decades have seen major efforts made to close gaps in health and social determinants of health for Indigenous persons. We ask whether relative progress toward these goals has been achieved.

**Methods:**

We used census data for each country to compare outcomes for the cohort aged 25–29 years at each census year 1981–2006 in the domains of education, employment, and income.

**Results:**

The percentage-point gaps between Indigenous and non-Indigenous persons holding a bachelor degree or higher qualification ranged from 6.6% (New Zealand) to 10.9% (Canada) in 1981, and grew wider over the period to range from 19.5% (New Zealand) to 25.2% (Australia) in 2006. The unemployment rate gap ranged from 5.4% (Canada) to 16.9% (Australia) in 1981, and fluctuated over the period to range from 6.6% (Canada) to 11.0% (Australia) in 2006. Median Indigenous income as a proportion of non-Indigenous median income (whereby parity = 100%) ranged from 77.2% (New Zealand) to 45.2% (Australia) in 1981, and improved slightly over the period to range from 80.9% (Canada) to 54.4% (Australia) in 2006.

**Conclusions:**

Australia, Canada, and New Zealand represent nations with some of the highest levels of human development in the world. Relative to their non-Indigenous populations, their Indigenous populations were almost as disadvantaged in 2006 as they were in 1981 in the employment and income domains, and more disadvantaged in the education domain. New approaches for closing gaps in social determinants of health are required if progress on achieving equity is to improve.

## Background

Indigenous peoples around the world experience higher rates of poor health, poverty, poor diet, inadequate housing and other social and health problems relative to non-Indigenous people. These disparities are found in nearly all countries with Indigenous populations, including some of the wealthiest nations in the Organisation for Economic Co-operation and Development (OECD) [1,2]. The narrowing of these gaps in health and socio-economic outcomes has been a focus of successive governments in these nations since at least the 1970s.

Understanding the complex historical, political and socio-economic factors that have led to the present situation has also been a key focus for medical and social sciences across the past four decades [1,2]. High-profile reviews published by the United Nations and others in recent years have documented the common factors underlying the continuation of health and social inequalities experienced by Indigenous populations across the globe, including systematic loss of culture and language, dispossession from traditional territories, and economic and social marginalization [2-4].

Indigenous inequality is a global health problem, but it is perhaps most surprising to witness its continuation in some of the world’s most wealthy countries. A commonly used barometer for the comparison of health and socio-economic development across countries is the United Nations' *Human Development Index* (HDI). Australia, Canada and New Zealand regularly place among the top 10 countries in the world on this annual measure, which combines education, income and life expectancy [5]. A previous study showed that these countries’ Indigenous populations would rank far lower on the HDI league table than their total populations, revealing the relative disadvantage of Indigenous peoples [6]. Each of these countries has since demonstrated a commitment to improving outcomes for Indigenous peoples by signing the United Nations *Declaration on the Rights of Indigenous Peoples*[7], which specifically articulates Indigenous peoples’ rights to “improvement of their economic and social conditions”.

The work of Marmot and others has demonstrated the existence of marked social gradients in health among the populations of wealthy nations [8]. In some cases the poorest groups in these societies have health and life-expectancy profiles similar to those living in developing nations. Much of this observed discrepancy in health outcomes has been attributed to so-called “social determinants of health”, which we might define as those non-health indicators of life outcomes which influence an individual’s health status across their life course. These can be socio-economic indicators such as education, employment status (including job type for those who are employed), income and wealth, property rights, justice system contacts, and social connections and supports, which impact a person’s ability to: obtain preventive health knowledge; apply that knowledge to their own life; and access appropriate health services when treatment is required for a given condition.

Marmot’s observations around health outcomes for the poor in relation to the unequal distribution of resources in wealthy societies [8] have been placed into global Indigenous perspective by the work of Gracey, King and Smith [3,4]. Where Marmot suggests that improving education, employment and income among disadvantaged segments of society will have positive implications for health and general wellbeing [8], Gracey, King and Smith [3,4] point out that the health of Indigenous populations may also be affected by additional and unique factors, such as cultural security, connection to lands, language, and culturally defined notions of health and wellbeing [3,4].

Our focus is on Australia, Canada, and New Zealand. In 2006 the combined Indigenous populations for these developed nations was 2.7 million persons, from a total population of about 55 million people [9-11]. These countries share a common pattern of mainly British colonization over their Indigenous populations; however important factors have uniquely shaped Indigenous-settler relations in each. These include: geography; the relative size of Indigenous and settler populations; and, in Canada, the influence of other colonial powers [12]. Despite these differences, persistent social, economic, and health disparities between Indigenous and non-Indigenous populations exist in all three countries.

Drawing on these perspectives, our study documents the relative progress made toward reaching equitable levels of socio-economic development among Indigenous citizens in Australia, Canada and New Zealand from 1981–2006, and looks at prospects for closing gaps in social determinants of health with non-Indigenous citizens in the coming 25 years. We focus on relative inequality in the human development domains of education, employment, and income, specifically among those aged 25 to 29 years. This is the age range by which most higher education has been completed, allowing us to more clearly see changes in educational attainment patterns. It is also the age by which a number of other important transitions have generally taken place, such as leaving the parental home, the transition from school to work, and the commencement of family formation, which have life-long implications for wellbeing and intergenerational transfers of human capability. Indeed, “closing the gap” likely requires particular attention to young people, and to the quality of these transitions. We believe this is the first time one study has brought together long-term data comparing these social determinants of health in the Indigenous populations of these three nations.

## Methods

### Study design

This study reports results from an analysis of census data for Australia, Canada, and New Zealand. Census data were used in preference to other data sources because of: the long time series available; consistency in measurement of questions and concepts over time; the availability of data for the same time points for each country; the absence of sample size issues; and the coverage of both Indigenous and non-Indigenous populations. Any effects on Indigenous wellbeing of the recent global slowdown in economic activity are not represented, as 2006 is the most recent census year for which these data are available for comparison between all three countries.

We measured progress of Indigenous persons aged 25–29 years relative to non-Indigenous persons aged 25–29 years over a 25 year period and across three human development domains: education; employment; and income. Information to support this investigation was obtained from the national statistics agencies of Australia, Canada and New Zealand for the census years 1981, 1986, 1991, 1996, 2001, and 2006, covering each domain of interest [13-15].

#### Indigenous populations

Australia, Canada and New Zealand have all included questions in their population censuses to identify their Indigenous populations in each of the years 1981–2006. This has allowed the data for each of the domains examined in the analyses to be disaggregated by Indigenous status for the three countries.

The term “Indigenous persons” is used interchangeably to refer to Australian Aboriginal and Torres Strait Islander peoples, Canadian Aboriginal peoples (including First Nations, Inuit and Métis), and New Zealand Māori.

#### Data access and permissions

Census data for Australia, Canada, and New Zealand were available to the authors via custom tabulations from their respective national statistical agencies. No special permissions or ethics committee approvals were required for this study as all research was undertaken using publically available de-identified and confidentialised data, ensuring the anonymity of all persons represented by the data.

### Measures

#### Education domain

Our measure was the proportion of Indigenous and non-Indigenous persons aged 25–29 years who had achieved a highest qualification of ‘bachelor degree or above’ in each of the census years 1981–2006 for each country.

‘Bachelor degree or above’ includes bachelor degrees, plus all postgraduate degrees, graduate diplomas and graduate certificates that require a completed bachelor degree as a pre-requisite for enrollment. While there are some differences in the way overall education statistics have been classified on the census forms of the three countries, there is very good comparability across all three countries for the classification ‘bachelor degree or above’ used by our study.

### Australia

The Australian Bureau of Statistics (ABS) provided us with a set of customized data tables from the Census of Population and Housing showing ‘highest level of qualification’ by Indigenous status for persons aged 25–29 years, calculated for all census years 1981 to 2006. We report data from these tables on persons with a classification of ‘bachelor degree or above’.

‘Highest level of qualification’ is derived from responses to census questions on the highest year of school completed and level of highest non-school qualification. The data excluded overseas visitors for all years [13].

### Canada

Statistics Canada provided us with a set of customized data tables from the Census of Population showing ‘highest level of schooling’ by Aboriginal designation, for persons aged 25–29 years, calculated for all census years 1981–2001, and ‘highest degree, certificate or diploma’ for 2006 [14].

The data refer to the highest grade or year of elementary or secondary school attended, or the highest year of university or other non-university education completed. University education is considered to be above other non-university education. Also, the attainment of a degree, certificate or diploma is considered to be at a higher level than years completed or attended without an educational qualification. From this data we were able to calculate the proportion of Aboriginal and non-Aboriginal persons aged 25–29 years who had achieved a highest qualification of ‘bachelor degree or above’ in each of the census years.

### New Zealand

Statistics New Zealand provided us with a set of customized data tables from the Census of Population and Dwellings showing ‘highest qualification’ by Māori ethnic group for persons aged 25–29 years, calculated for all census years 1981 to 2006 [15].

'Highest qualification' is derived for people aged 15 years and over, and combines responses to census questions on the highest secondary school qualification and post-school qualification, to derive a single highest qualification. The output categories prioritize post school qualifications over any qualification received at school. From this data we were able to calculate the proportion of Māori and non- Māori persons aged 25–29 years who had achieved a highest qualification of ‘bachelor degree or above’ in each of the census years.

#### Labour force domain

Our measure was a census derived unemployment rate for each country. The census labour force variables were consistent for all three countries, with classifications of ‘employed’, ‘unemployed’ and ‘not in the labour force’ provided via custom tables from the statistical agencies of each country [13-15].

A person is said to be ‘unemployed’ if they had no job in the past week but were actively looking for work. A person is regarded as being ‘in the labour force’ if they are currently employed or actively looking for work. Persons in neither category are regarded as being ‘not in the labour force’ and are not included in unemployment calculations.

Unemployment rates were produced for the Indigenous and non-Indigenous populations for each country using the following calculation:

$$\mathrm{Unemployment}\;\mathrm{rate}=\frac{\mathrm{unemployed}\;\mathrm{persons}\;}{\mathrm{persons}\;\mathrm{in}\;\mathrm{the}\;\mathrm{labour}\;\mathrm{force}}\times 100$$

Additionally there had been little change in the categories of labour force status at the broad level across the six censuses for any of the countries, making this variable suitable for analysis across multiple time points.

#### Income domain

Our measure was median Indigenous personal income as a proportion of median non-Indigenous personal income in each of the census years for each country.

The information on annual personal median incomes for persons aged 25–29 years for each census year for Australia, Canada, and New Zealand was sourced from the statistical agency of each country [13-15].

## Results

**Figure 1:**
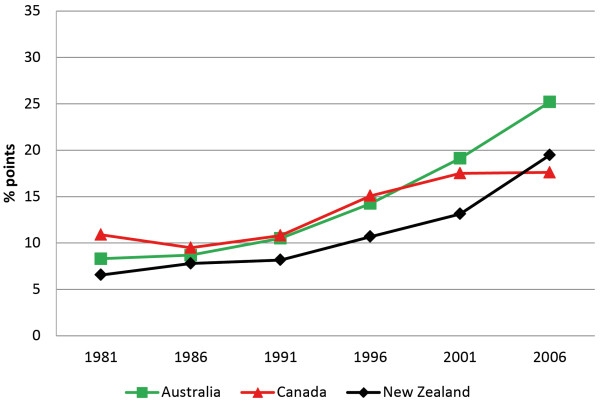
**Bachelor degree or above gap — Persons aged 25-29 years.** The percentage point gap between the proportion of Indigenous and non-Indigenous persons aged 25–29 years recorded as having a qualification of bachelor degree or above on the census. A gap of zero equates to parity.

For the indicator ‘the proportion of those with a bachelor degree or higher qualification’ the gaps in all countries were wide, and in fact grew wider over the period (Figure 1). For example, in Australia for those aged 25 to 29 years the gap rose from 8 to 25 percentage points between 1981 and 2006. Australia clearly fared the worst of the three countries in terms of the increase in the gap for this indicator, but even the best performer, Canada, showed a gap of 17.6 percentage points by 2006. This is not to say that educational outcomes for Indigenous people have worsened. The data for all three countries clearly indicate absolute gains in the proportion of Indigenous people with bachelor degree or higher qualifications (Table 1). However, in relative terms Indigenous people were increasingly behind the non-Indigenous populations on this measure.

**Table 1:** Persons aged 25–29 years on the census: Indigenous vs. non-Indigenous population level outcomes across three measures of socio-economic development from 1981–2006 for Australia, Canada and New Zealand

	**Australia**					
	**1981**	**1986**	**1991**	**1996**	**2001**	**2006**
**Bachelor degree and above (%)**						
Indigenous	0.3	0.5	1.1	2.6	3.5	4.1
Non-Indigenous	8.6	9.2	11.6	16.8	22.6	29.3
Gap	8.3	8.7	10.5	14.2	19.1	25.2
**Unemployment rate (%)**						
Indigenous	22.3	33.1	31.5	23.2	21.9	16.3
Non-Indigenous	5.5	9.3	12.3	9.3	7.7	5.3
Gap	16.9	23.8	19.3	13.8	14.2	11.0
**Median individual income ($Aus)**						
Indigenous	4,643	8,115	11,032	12,376	15,236	18,824
Non-Indigenous	10,273	14,928	20,224	22,932	28,340	34,632
Indigenous % of non-Indigenous	45.2	54.4	54.5	54.0	53.8	54.4
**Population aged 25-29 (no.)**						
Indigenous	12,200	19,500	23,100	30,600	32,600	30,800
Non-Indigenous	1,127,000	1,249,000	1,272,000	1,288,000	1,237,000	1,164,000
	**Canada**					
	**1981**	**1986**	**1991**	**1996**	**2001**	**2006**
**Bachelor degree and above (%)**						
Aboriginal	3.4	4.5	6.2	7.1	9.0	11.4
Non-Aboriginal	14.3	14.0	17.0	22.2	26.5	29.0
Gap	10.9	9.5	10.8	15.1	17.5	17.6
**Unemployment rate (%)**						
Aboriginal	14.8	22.4	21.7	22.8	17.1	13.3
Non-Aboriginal	7.3	10.8	11.5	10.7	7.6	6.7
Gap	7.4	11.6	10.2	12.1	9.5	6.6
**Median individual income ($Can)**						
Aboriginal	7,666	10,953	14,488	13,218	16,391	19,507
Non-Aboriginal	12,712	16,090	20,872	19,805	23,912	25,644
Aboriginal % of non-Aboriginal	60.3	68.1	69.4	66.7	68.5	76.1
**Population aged 25-29 (no.)**						
Aboriginal	41,800	71,100	99,200	96,800	105,000	125,000
Non-Aboriginal	2,124,000	2,256,000	2,259,000	1,926,000	1,782,000	1,851,000
	**New Zealand**					
	**1981**	**1986**	**1991**	**1996**	**2001**	**2006**
**Bachelor degree and above (%)**						
Māori	1.2	1.8	2.2	3.9	7.0	10.0
Non-Māori	7.7	9.5	10.4	14.6	20.1	29.5
Gap	6.6	7.8	8.2	10.7	13.1	19.5
**Unemployment rate (%)**						
Māori	8.7	13.7	26.7	18.7	17.5	11.6
Non-Māori	3.4	5.3	10.0	6.7	6.8	4.8
Gap	5.4	8.4	16.7	11.9	10.7	6.8
**Median individual income ($NZ)**						
Māori	7,100	11,200	13,100	15,500	18,500	25,400
Non-Māori	9,200	14,700	20,300	23,400	26,200	31,400
Māori% of non-Māori	77.2	76.2	64.5	66.2	70.6	80.9
**Population aged 25-29 (no.)**						
Māori	29,300	36,100	39,600	43,100	40,200	38,100
Non-Māori	207,000	228,000	230,000	217,000	196,000	192,000

**Figure 2:**
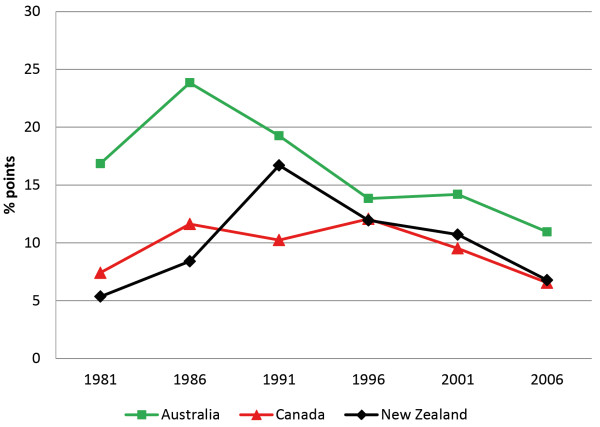
**Unemployment rate gap — Persons aged 25-29 years.** The percentage point gap between the proportion of Indigenous and non-Indigenous persons aged 25–29 years who were recorded as unemployed on the census. A gap of zero equates to parity. Note that for this period Australia had in place a program whereby Indigenous persons could work for unemployment benefits (Community Development Employment Projects – CDEP), and doing so has meant being recorded as “employed” on official labor force statistics, reducing Indigenous unemployment and potentially distorting the true gap [16].

While Indigenous people had consistently higher unemployment, there was fluctuation in the unemployment rate gap over the period 1981 to 2006 for all three countries (Figure 2). By 2006, both Australia and Canada showed a narrower gap than that observed in 1981, while the gap for New Zealand had widened slightly. However, Australia maintained the widest unemployment rate gap of the three countries over the entire period, despite the gap reducing from 16.9 to 11.0 percentage points. Canada finished the period with the narrowest gap (6.6 percentage points).

**Figure 3:**
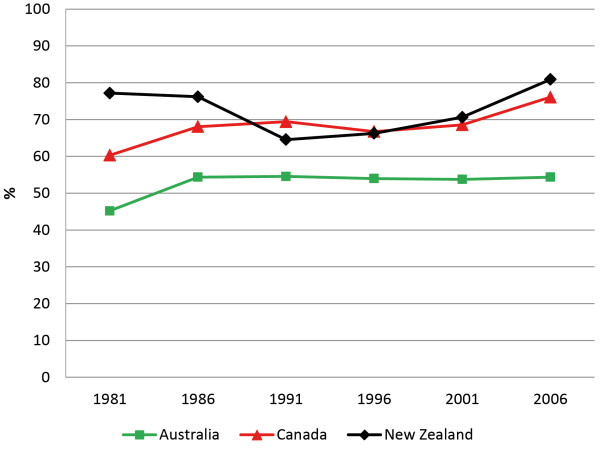
**Indigenous income as a percentage of non-Indigenous income — Annual personal median income, persons aged 25-29 years.** Median Indigenous income as a proportion of non-Indigenous median income, for persons aged 25–29 years on the census. Parity equals 100%.

Median Indigenous income as a proportion of non-Indigenous median income (whereby parity = 100%) ranged from 77.2% (New Zealand) to 45.2% (Australia) in 1981, and improved slightly over the period to range from 80.9% (Canada) to 54.4% (Australia) in 2006. Overall, the gap remained steady for Australia, while for Canada and New Zealand there was some fluctuation over the period (Figure 3). Again, Australia fared the worst, with Indigenous median annual income barely reaching above half that of non-Indigenous people across the reference period, while Canada and New Zealand had made some improvements by 2006.

## Discussion

Wealthy developed nations with a colonial past, such as Australia, Canada, and New Zealand, have typically under-resourced the human development of their Indigenous populations for much of their post-colonial histories. Impact has been felt across most aspects of Indigenous life, including health, education, participation in the economy, legal rights to traditional lands and resources, cultural security, and wider issues of social inclusion. Though government mandated reparations have been in place since at least the 1970s, long standing inequality has left the Indigenous peoples of these countries behind their non-Indigenous counterparts on indicators of health, wealth, social justice, and general wellbeing [2]. This research comparing social determinants of health for Australia, Canada, and New Zealand, suggests that such inequalities have persisted—in some cases barely improving across 25 years, with Australia the worst performer overall—despite concerted efforts by governments to close gaps in outcomes for Indigenous people in recent decades. These countries are now challenged with finding new approaches to solving this social inequality issue, if health and socio-economic conditions for Indigenous people are to even approach parity with non-Indigenous persons within a generation.

The social determinants of health observed in this study covered educational attainment, labour force activity and income. We specifically examined the gaps between Indigenous and non-Indigenous people using the proportion with a bachelor’s degree or higher, unemployment rates, and median annual income. There are other indicators of “wellbeing” upon which these populations could be compared. However, people’s connection to the labour force, higher formal educational attainment and income are critical aspects of participation and inclusion in these societies, and key social determinants of health. In the terms of Nobel laureate and HDI author Amartya Sen, being engaged in work and having sufficient income represent “functionings” that help one to make meaningful life choices in order to realize “capabilities” [17]. In the context of advanced economies, these capabilities have direct implications for wellbeing.

While a persistent gap exists between Indigenous and non-Indigenous outcomes for these indicators, we hypothesized that this gap should have narrowed over time. Our results show that in absolute terms there was some improvement on all three indicators for all three countries, but no consistent narrowing of the relative gaps for any country (Figures 1, 2 and 3). As Table 1 shows, reductions in the indicator gaps for some time periods are due to fluctuation in the measures for non-Indigenous people, as opposed to improvements for Indigenous people.

The increasing gap in educational attainment is largely due to rapid increases in the proportion of university qualified young people in the non-Indigenous populations of all three countries (Table 1). This expansion in higher education is closely linked to compositional shifts in developed economies away from manufacturing and into knowledge-based service industries, and each of these countries has experienced periods of macro-economic restructuring towards a more knowledge based economy [18]. As relatively fewer Indigenous people complete university education, they are largely excluded from this sector of the economy. With education becoming an increasingly critical component to accessing the employment and income benefits of advanced modern economies, the effects of these compositional changes may have offset any gains from social policy investments in closing socio-economic gaps.

Reducing these gaps means addressing a complex set of issues. Increasing educational attainment requires appropriately resourced education support beginning in early childhood, sustained throughout regular schooling and into vocational and higher education settings. These programs should support Indigenous peoples’ aspirations, including the maintenance of cultural integrity [19]. Factors beyond the school gate that support Indigenous engagement with the education process are also critical. We know that pathways to disadvantage in education begin in the early years, with high proportions of Indigenous children already behind their non-Indigenous peers in academic performance from their first year in school—a deficit that continues throughout primary and high school [20]. Higher rates of school absenteeism and lower levels of parental education may contribute to the widening disparity in academic performance over time for Indigenous children, and the resources and role models for scholastic learning that often exist in non-Indigenous homes may be largely absent in many Indigenous households [21].

Given these trends, closing the higher education gap between Indigenous and non-Indigenous young people will require a major change in policy approach, and patience. It must be recognized that changes made today to improve young people’s readiness for school will take years to result in higher rates of university completion. This suggests that flow-on effects of higher employment and incomes may be even further away. Any suggestion that gaps in socioeconomic outcomes can be eliminated in the near future seems unrealistic. Without significant increases in the proportion of young Indigenous people completing higher education, these gaps will remain indefinitely. In developed economies, population-wide improvements in income are mostly related to improvements in educational achievement and opportunities for employment. Our study suggests both Canada and New Zealand are starting to improve income disparity issues for their Indigenous people, though each is still some way from achieving parity. The situation for Indigenous Australians is far less encouraging.

The health and wellbeing of Indigenous populations in these countries has been a key aspect of national public policy for some time. In addition to important legal changes regarding the recognition of traditional rights, governments have engaged in various efforts to improve conditions for Indigenous peoples, including education, health and employment programmes, and policy changes. For example, most recently, the government of Australia has made “closing the gap” in human development outcomes between Indigenous and non-Indigenous people an explicit goal of national policy [22], and the Government of Canada and Assembly of First Nations’ *Joint Action Plan* has a focus on increasing access to education and employment opportunity [23], while New Zealand has used a “closing the gaps” theme for policies aimed at social justice issues for Māori [24]. Adding to this already complex policy environment is the observation that these countries have seen some growth in Indigenous populations across the reference period, in addition to that from births, due to changing patterns of self-identification in their census [25-27].

### Limitations

There are several limitations to the methodology employed in this study. It is known that across time there has been a change in the propensity of people to identify as Indigenous in all three countries [25-27]. This means, for example, that the composition of the Indigenous population of 1981 is likely different to that of 2006 for all age groups, which may have influenced some of the results seen in this study. Another issue is that the scope of national census questions may be too limited to explain some of the differences in outcomes between Indigenous and non-Indigenous persons. For example, there may be sound cultural reasons for why an Indigenous person does not seek to participate in certain educational or employment spheres, but we can’t measure that with census data. Lastly, as census data are only gathered once every five years we are unable to track economic and social change as closely as something like a longitudinal survey with annual follow-up.

## Conclusions

Australia, Canada, and New Zealand represent nations with some of the highest levels of human development in the world, yet our research shows that their Indigenous populations were almost as disadvantaged in 2006 as they were in 1981, relative to their non-Indigenous populations, on three key social determinants of health. These ongoing disparities represent a major public policy concern, and a growing focus for science and human rights organizations. Given the breadth of scientific inquiry, the public spending and good intentions of successive Australian, Canadian and New Zealand governments regarding Indigenous health and social advancement since 1981, the fact that relative progress on key social determinants of health has been practically static for Indigenous peoples is alarming. Despite absolute improvements on these indicators, continuing disparities suggest that existing approaches to addressing Indigenous inequality are not as effective they need to be. They also suggest that achieving equity may take several more decades, especially as the young adult populations described here are the ones in which more progress was expected to have occurred across these domains. Surely Indigenous peoples in these nations would be within their rights to expect a narrowing of these gaps to occur over the coming 25 years, along with improvements in health outcomes. Science and policy are yet to provide viable solutions to this enduring social equity issue. If “closing the gap” in health and socio-economic disparity between Indigenous and non-Indigenous people remains a goal, it would seem that completely new approaches are required to achieve success, otherwise Indigenous persons in these developed nations are being consigned to a future of entrenched inequality for generations to come.

## Competing interests

The authors declare that they have no competing interests.

## Authors’ contributions

FM and MC had the original idea for the study, developed the analytic concept, and acquired the data. DP and EM compiled the data and performed the analysis. FM, MC and DP wrote the first draft. DL, EG, and SRZ contributed to all subsequent drafts and revisions. All authors read and approved the final manuscript.

## Pre-publication history

The pre-publication history for this paper can be accessed here:

http://www.biomedcentral.com/1471-2458/14/201/prepub
